# Impact of Macrophage Inflammatory Protein-1α Deficiency on Atherosclerotic Lesion Formation, Hepatic Steatosis, and Adipose Tissue Expansion

**DOI:** 10.1371/journal.pone.0031508

**Published:** 2012-02-16

**Authors:** Arion Kennedy, Marnie L. Gruen, Dario A. Gutierrez, Bonnie K. Surmi, Jeb S. Orr, Corey D. Webb, Alyssa H. Hasty

**Affiliations:** Department of Molecular Physiology and Biophysics, Vanderbilt University School of Medicine, Nashville, Tennessee, United States of America; Università degli Studi di Milano, Italy

## Abstract

Macrophage inflammatory protein-1α (CCL3) plays a well-known role in infectious and viral diseases; however, its contribution to atherosclerotic lesion formation and lipid metabolism has not been determined. Low density lipoprotein receptor deficient (LDLR^−/−^) mice were transplanted with bone marrow from CCL3^−/−^ or C57BL/6 wild type donors. After 6 and 12 weeks on western diet (WD), recipients of CCL3^−/−^ marrow demonstrated lower plasma cholesterol and triglyceride concentrations compared to recipients of C57BL/6 marrow. Atherosclerotic lesion area was significantly lower in female CCL3^−/−^ recipients after 6 weeks and in male CCL3^−/−^ recipients after 12 weeks of WD feeding (*P*<0.05). Surprisingly, male CCL3^−/−^ recipients had a 50% decrease in adipose tissue mass after WD-feeding, and plasma insulin, and leptin levels were also significantly lower. These results were specific to CCL3, as LDLR^−/−^ recipients of monocyte chemoattractant protein^−/−^ (CCL2) marrow were not protected from the metabolic consequences of high fat feeding. Despite these improvements in LDLR^−/−^ recipients of CCL3^−/−^ marrow in the bone marrow transplantation (BMT) model, double knockout mice, globally deficient in both proteins, did not have decreased body weight, plasma lipids, or atherosclerosis compared with LDLR^−/−^ controls. Finally, there were no differences in myeloid progenitors or leukocyte populations, indicating that changes in body weight and plasma lipids in CCL3^−/−^ recipients was not due to differences in hematopoiesis. Taken together, these data implicate a role for CCL3 in lipid metabolism in hyperlipidemic mice following hematopoietic reconstitution.

## Introduction

Atherosclerotic lesion formation is a multifaceted disease process, and immune cell infiltration into the artery wall is an essential component of lesion growth. Macrophages are one of the predominant cell types that are recruited to the artery wall; however, other immune cells such as T-cells and neutrophils can also enter atherosclerotic lesions. Many different factors contribute to leukocyte recruitment to the artery wall and the best understood are chemoattractant cytokines called chemokines. Chemokines are 8–10 kDa proteins that bind to receptors such as the CC and CXC family of G-protein coupled receptors. One of the most potent macrophage chemokines is monocyte chemoattractant protein −1 (CCL2). Mice with a global deletion of CCL2 or its receptor, CCR2, demonstrate protection from atherosclerotic lesion formation [1], [2], [3].

Another chemokine, macrophage inflammatory protein-1α, CCL3 in standard nomenclature, has been shown to be increased in the aortas of apolipoprotein E deficient (apoE^−/−^) mice [4] and in human carotid endarterectomy samples [5]. It is a member of the CC chemokine family and found in the same subfamily as CCL4, CCL5, and CCL15. Like most chemokines, CCL3 is a chemoattractant for several different leukocytes, with varying degrees of potency (monocytes = T-cells>neutrophils>eosinophils). CCL3 expression is inducible in most mature hematopoietic cells by LPS, viral infection, and TNFα [6], [7]. Our laboratory has also shown that macrophage CCL3 expression is dramatically induced by incubation with VLDL [8]. Thus, CCL3 may impact atherosclerotic lesion formation via several different mechanisms.

Despite the known importance of chemokines in atherosclerotic lesion formation and the extensive literature on CCL2, little is known regarding the role of CCL3 in atherosclerosis or in other metabolic processes. Hyperlipidemia is positively associated with the production of CCL3 in a various mouse models of atherosclerosis and obesity [9], [10], [11]. Similarly, CCL3 has been shown to be elevated in the plasma and metabolic tissues (liver and adipose tissue) of patients with hyperlipidemia and metabolic disease [12], [13]. Based on these associations and the function of CCL3, it is possible that under hyperlipidemic conditions, CCL3 is an important chemokine in recruitment of immune cells to atherosclerotic lesions and other metabolic tissues.

In this study, we sought to determine whether absence of CCL3 expression could protect against atherosclerotic lesion formation in LDLR^−/−^ mice. To this end, we performed bone marrow transplantation (BMT) studies in LDLR^−/−^ recipient mice and also developed CCL3^−/−^;LDLR^−/−^ double knockout mice. Our findings demonstrate that hematopoietic CCL3 plays a role in atherosclerotic lesion formation by regulating lipid metabolism.

## Results

### STUDY 1

#### Plasma lipids and insulin concentrations are lower in CCL3^−/−^→LDLR^−/−^ mice

Male and female LDLR^−/−^ mice were lethally irradiated and transplanted with bone marrow from C57BL/6 or CCL3^−/−^ donor mice. At 4 weeks post-BMT, mice were placed on WD for 6 or 12 weeks (Study #1 in Figure S1). No differences in total cholesterol (TC) or triglyceride (TG) levels between the two transplant recipient groups were observed before transplantation (Table 1), although non esterified fatty acid (NEFA) levels were higher in recipients of CCL3^−/−^ marrow. After 6 weeks of WD feeding, plasma TC and TG were increased from baseline in all mice. However, in both male and female mice, the recipients of CCL3^−/−^ marrow had significantly lower TC (22–24%, *P*<0.01), TG (32–38%, *P*<0.001), and NEFA (19–20%, *P*<0.05) compared to the C57BL/6 recipients. Plasma insulin levels were also lower in male CCL3^−/−^→LDLR^−/−^ mice (50%, *P*<0.0005). A glucose tolerance test in a subset of mice did not reveal improvements in glucose tolerance, although plasma insulin levels trended to be reduced 30 min after administration of the glucose bolus (Figure S2). By 12 weeks post-WD feeding, TC and TG remained significantly lower in the female CCL3^−/−^→LDLR^−/−^ mice compared to the C57BL/6→LDLR^−/−^ controls and in male mice, there was a trend for a reduction in TGs and NEFAs. FPLC analysis revealed that the reduction in plasma lipids were due to modest decreases in VLDL and LDL cholesterol (Figure S3).

**Table 1:** Plasma parameters in CCL3^−/−^→LDLR^−/−^ mice.

Donor Marrow	n	TC (mg/dL)	TG (mg/dL)	NEFA (mEq/L)	Insulin (ng/mL)	Leptin (ng/mL)
			Baseline			
**Male**						
C57BL/6	28	125±4	92±4	0.89±0.06	N/D	N/D
CCL3^−/−^	27	128±4	99±6	1.29±0.08∥	N/D	N/D
**Female**						
C57BL/6	18	149±9	81±6	1.04±0.10	N/D	N/D
CCL3^−/−^	21	134±7	79±4	1.11±0.09	N/D	N/D

			6 weeks post-WD			
**Male**						
C57BL/6	15	793±33	270±18	0.91±0.05	0.72±0.05	8.96±0.85
CCL3^−/−^	14	624±47†	167±11#	0.74±0.05*	0.41±0.05∥	4.45±0.58∥
**Female**						
C57BL/6	11	833±33	236±12	0.86±0.04	0.54±0.05	9.05±1.68
CCL3^−/−^	12	639±43‡	160±15§	0.69±0.03‡	0.42±0.05	5.70±1.49

			12 weeks post-WD			
**Male**						
C57BL/6	13	634±34	364±58	1.39±0.14	0.90±0.08	17.75±2.75
CCL3^−/−^	13	587±34	278±34	1.11±0.86	0.70±0.07	7.45±1.69‡
**Female**						
C57BL/6	7	687±41	227±14	1.05±0.09	0.71±0.07	11.28±2.27
CCL3^−/−^	9	508±32‡	160±12‡	0.97±0.11	0.69±0.06	7.26±1.46

LDLR^−/−^ mice were transplanted with C57BL/6 or CCL3^−/−^ bone marrow. At 4 weeks post-BMT, mice were placed on WD for either 6 or 12 weeks. Blood was collected for plasma analysis before transplantation (baseline) and at 6 and 12 weeks post-WD. For baseline plasma analysis, values were not different between the two transplant groups; thus, these cohorts are combined in the table. Six week and 12 week values represent only the mice sacrificed at that time point. Data are the mean ± SEM. N/D = not determined.

* *P*<0.05.

† *P*<0.01.

‡ *P*<0.005.

§ *P*<0.001.

∥ *P*<0.0005.

# *P*<0.00001.

#### Atherosclerotic lesion area is reduced in CCL3^−/−^→LDLR^−/−^ mice

**Figure 1:**
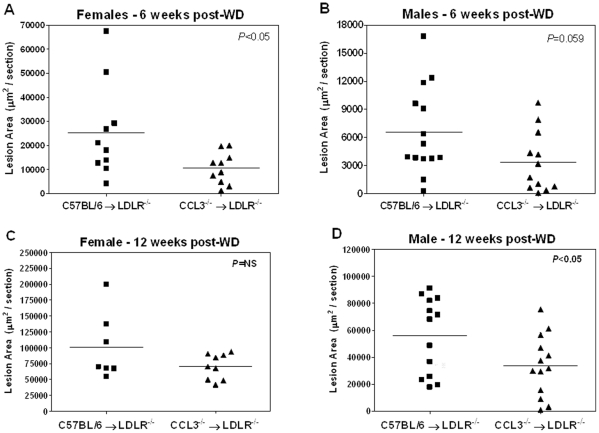
Recipients of CCL3^−/−^ bone marrow have reduced lesion area. LDLR^−/−^ mice were transplanted with C57BL/6 or CCL3^−/−^ bone marrow according to the Methods section. At 4 weeks post-BMT, mice were placed on WD for 6 or 12 weeks. Aortic root lesion area was quantified according to the Methods section. Panels A and B show lesion area from female and male mice after 6 weeks of WD feeding, respectively. Data are presented as mean ± SEM from 7–14 mice per group. Panels C and D show the lesion area from female and male mice after 12 weeks of WD feeding, respectively. Data are mean ± SEM from 7–13 mice per group.

**Figure 2:**
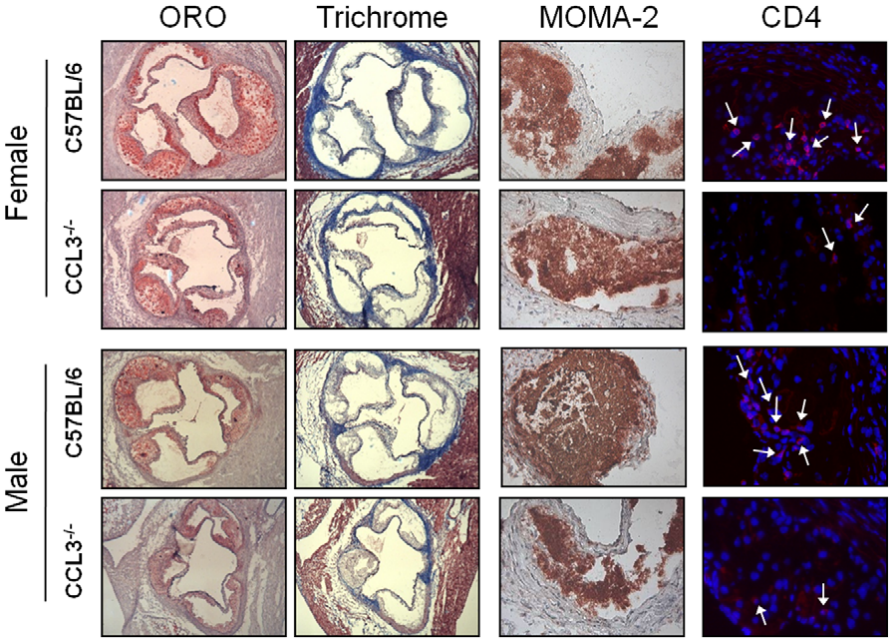
Lesion complexity does not differ with CCL3 bone marrow genotype. LDLR^−/−^ mice were transplanted with C57BL/6 or CCL3^−/−^ bone marrow. At four weeks post-BMT, mice were placed on WD for 12 weeks. ORO (4×), Trichrome, MOMA-2, and CD4 stained lesion (10×) are in columns as indicated on figure. Gender and donor marrow are indicated on the left of the figure. Sections were chosen from images representing mice with lesion areas close to the mean of their respective groups.

**Figure 3:**
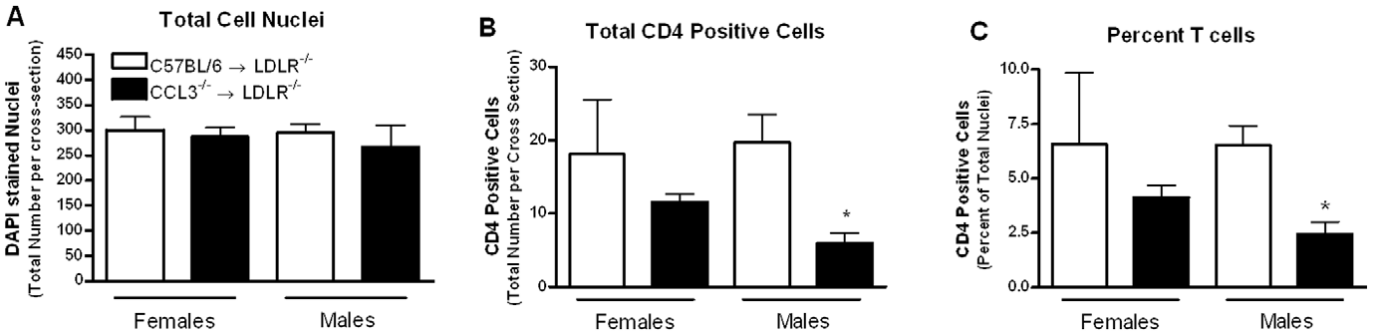
Recipients of CCL3^−/−^ marrow have reduced lesional T lymphocytes. Sections from the aortic root of male and female CCL3^+/+^→LDLR^−/−^ and CCL3^−/−^→LDLR^−/−^ mice were used for immunofluorescent analysis of CD4 positive T-cells according to the Methods section. For each mouse, data from the three valves was combined. Total dapi stained nuclei (Panel A) and CD4 positive cells were quantified (Panel B). The percent of cells that were CD4 T cells was calculated by dividing the total cells by the CD4 positive cells (Panel C). Data represent the mean ± SEM of 3–6 mice per group. * *P*<0.01 compared to male CCL3^+/+^→LDLR^−/−^.

Atherosclerotic lesion area was measured from ORO-stained sections in the aortic root of mice fed the WD for 6 and 12 weeks (Figs. 1 and 2). For both time points, female mice had larger lesions than male mice. After 6 weeks of WD feeding, female CCL3^−/−^→LDLR^−/−^ mice showed a significant decrease in lesion area compared to their controls (*P*<0.05; Figure 1A), while male CCL3^−/−^→LDLR^−/−^ mice showed a trend toward a reduction in aortic root lesion area compared to C57BL/6→LDLR^−/−^ controls (*P* = 0.059; Figure 1B). A trend toward decreased lesion area was detected in the 12 week WD fed female recipients of CCL3^−/−^ marrow compared to recipients of C57BL/6 marrow (Figure 1C). Aortic root lesion area was significantly reduced in male CCL3^−/−^→LDLR^−/−^ mice compared to C57BL/6→LDLR^−/−^ mice (*P*<0.05; Figure 1D). No differences in lesion complexity (Figure 2, Trichrome stained panels) were apparent. In addition, despite the differences in lesion area between the two recipient groups, in all cases, nearly the entire lesion stained positive for macrophages (Figure 2, MOMA-2 immunostained panels). Immunofluorescence staining for CD4 positive T cells demonstrated a significantly decreased number and percent of T cells in the CCL3^−/−^→LDLR^−/−^ male mice compared to controls (Figs. 2 and 3).

#### Body adiposity is lower in CCL3^−/−^→LDLR^−/−^ mice

**Figure 4:**
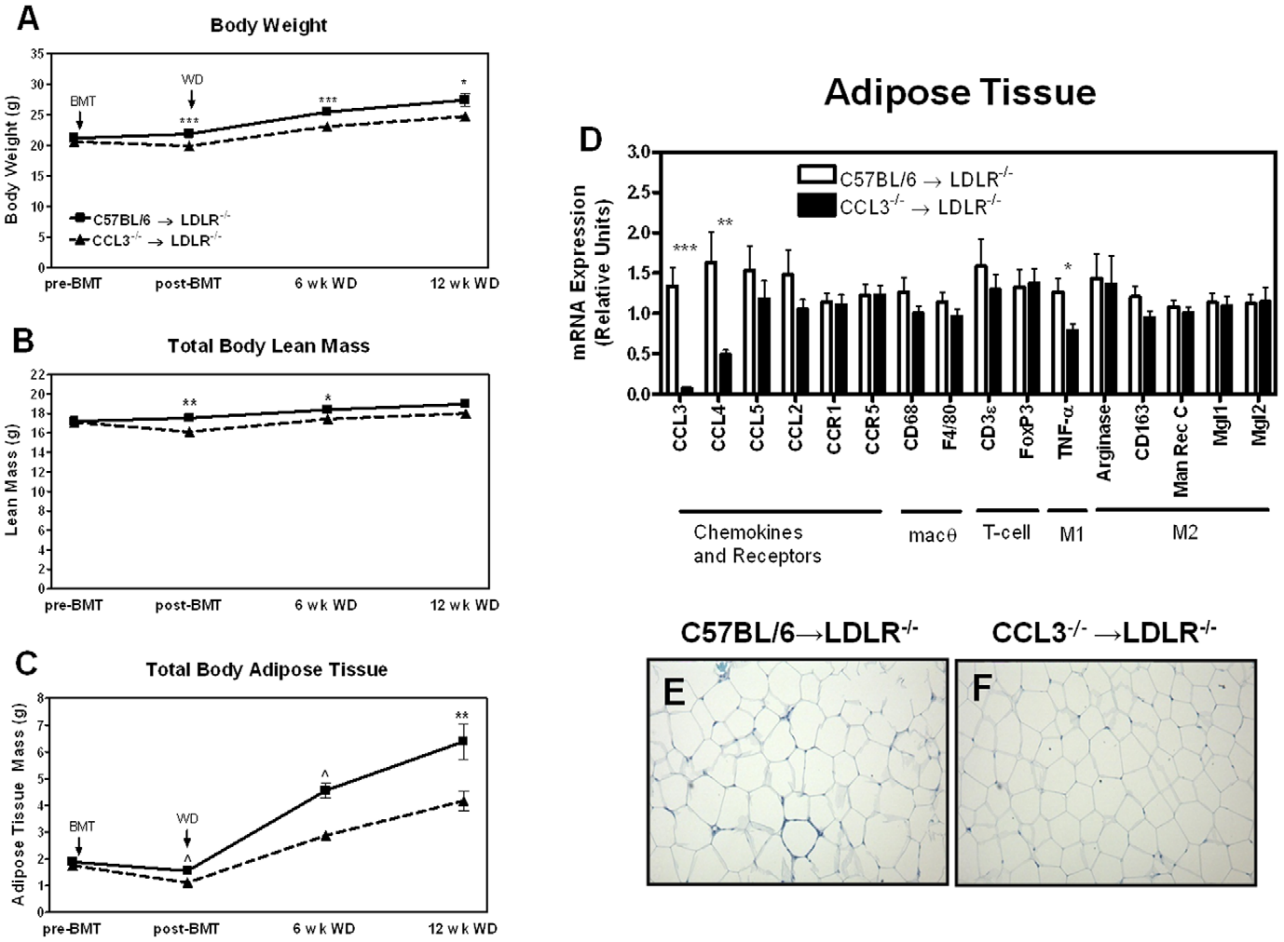
Adipose tissue mass and gene expression. Male LDLR^−/−^ mice were transplanted with C57BL/6 or CCL3^−/−^ bone marrow. At 4 weeks post-BMT, mice were placed on WD for either 6 or 12 weeks. (A–C) Body weight, total body lean mass, and total body adipose tissue were measured by NMR pre-BMT, 4 weeks post-BMT, and at 6 and 12 weeks post-WD. Data from pre-BMT, 4 weeks post-BMT, and 6 weeks post-WD are combined for the 6 week and 12 week groups. The 12 week post-WD data represent only the mice sacrificed at that time point. (D–F) Perigonadal adipose tissue was collected from the mice fed WD for 12 weeks. RNA was isolated and used for real-time RT-PCR analysis as described in the Methods section. Data are the mean ± SEM of the relative gene expression for 12–14 mice per group. Images are from Toluidine Blue O stained sections processed as described in the Methods section. * *P*<0.05 ** *P*<0.01 *** *P*<0.0005 CCL2 = monocyte chemoattractant protein-1 TNF-α = tumor necrosis factor-α Man Rec C = mannose receptor C Mgl = macrophage galactose N-acetyl-galactosamine specific lectin.

Body weight as well as total body fat and lean mass were measured one week pre-BMT, at 4 weeks post-BMT, and at 6 and 12 weeks post-WD (Table 2). There were no differences in total body weight, total fat mass, or total muscle mass in female mice between the two groups at any time point. However, in male mice, weight gain after WD-feeding was reduced in the CCL3^−/−^→LDLR^−/−^ compared to C57BL/6→LDLR^−/−^ mice at each of the three time points post-BMT (Figure 4A). This was reflected by a decrease in muscle mass at 4 weeks post-BMT and at 6 weeks post-WD (Figure 4B); however, the most significant difference was seen in total body adipose tissue mass (Figure 4C) where total body fat of the CCL3^−/−^ recipients was 56% lower at 12 weeks post-WD. At sacrifice, perigonadal fat pad mass of CCL3^−/−^→LDLR^−/−^ mice was 61% lower compared to C57BL/6→LDLR^−/−^ mice (*P*<0.01, Table 2). In addition, plasma leptin levels were 2.4-fold lower in the CCL3^−/−^ recipients (*P*<0.005, Table 1). The lower body weight and adipose tissue mass were not due to a general wasting, as the mice appeared healthy and the mass of other organs such as liver (Table 2) and kidney (data not shown) were not different between the groups.

**Table 2:** Body and tissue weights in CCL3^−/−^→LDLR^−/−^ mice.

Donor Marrow	n	Body Weight (g)	Total Adipose Tissue Mass (g)	Lean Tissue Mass (g)	Perigonadal Adipose Tissue Mass (g)	Liver Mass (g)
	Baseline					
**Male**						
C57BL/6	28	21.1±0.3	1.9±0.1	17.2±0.3	N/D	N/D
CCL3^−/−^	27	20.8±0.3	1.8±0.1	17.1±0.2	N/D	N/D
**Female**						
C57BL/6	18	17.2±0.3	2.2±0.1	13.6±0.3	N/D	N/D
CCL3^−/−^	21	16.8±0.3	2.0±0.1*	13.7±0.3	N/D	N/D

	6 weeks post-WD					
**Male**						
C57BL/6	15	24.4±0.6	4.2±0.3	17.9±0.4	0.67±0.04	1.10±0.03
CCL3^−/−^	14	22.7±0.4	2.7±0.2§	17.2±0.4	0.41±0.03∥	0.99±0.05
**Female**						
C57BL/6	11	19.6±0.3	3.1±0.3	14.6±0.2	0.35±0.03	1.01±0.03
CCL3^−/−^	12	18.5±0.3*	2.6±0.3	14.5±0.2	0.25±0.04	0.96±0.02

	12 weeks post-WD					
**Male**						
C57BL/6	13	27.5±1.0	6.4±0.7	19.0±0.4	1.08±0.11	1.26±0.08
CCL3^−/−^	13	24.8±0.5*	4.1±0.4†	18.0±0.3	0.67±0.06‡	1.10±0.04
**Female**						
C57BL/6	7	21.0±0.6	3.7±0.5	15.6±0.3	0.44±0.07	1.05±0.05
CCL3^−/−^	9	20.6±0.7	3.6±0.4	15.5±0.4	0.41±0.05	1.00±0.05

LDLR^−/−^ mice were transplanted with C57BL/6 or CCL3^−/−^ bone marrow. At 4 weeks post-BMT, mice were placed on WD for either 6 or 12 weeks. Body weight as well as total body lean and fat mass were measured at each time point. There were no differences in values between the two transplantations cohorts at baseline; thus, these values are presented for all mice together. Six week and 12 week values represent only the mice sacrificed at that time point. Data are the mean ± SEM. N/D = not determined.

*P<0.05.

† *P*<0.01.

‡ *P*<0.005.

§ *P*<0.0005.

∥ *P*<0.0001.

#### Gene expression in adipose tissue of CCL3^−/−^→LDLR^−/−^ mice

Because macrophage accumulation in adipose tissue is closely associated with the degree of adiposity [14], [15], and chemokines are thought to play a role in their recruitment to adipose tissue, we performed realtime RT-PCR analysis for markers of macrophage gene expression in RNA isolated from perigonadal fat pads (Figure 4D). Interestingly, CCL3 expression was nearly undetectable in the adipose tissue of CCL3^−/−^→LDLR^−/−^ mice. Expression of CCL2 was not different between groups; however, CCL4 was significantly lower in CCL3^−/−^ recipients (*P*<0.01). Expression of the CCL3 receptors, CCR1 and CCR5, was not different between the transplant groups. Similarly, expression of the macrophage markers F4/80 and CD68 and the T-cell markers CD3ε and FoxP3 were not different between the two BMT groups. To evaluate the polarization of macrophages within the adipose tissue, expression of M1 and M2 markers was quantified. The M1, pro-inflammatory cytokine TNF-α was significantly reduced (*P*<0.05) in CCL3^−/−^ recipients. M2 markers, arginase 1, CD163, mannose receptor C, Mgl1, and Mgl2 were not different. Toluidine Blue O of the adipose tissue revealed no obvious difference in adipocyte size between the two groups (Figure 4E–F). Similar gene expression patterns were found in the adipose tissue of female mice, although the M2 markers were significantly reduced in the CCL3^−/−^ recipients (data not shown).

#### Liver lipids and gene expression in CCL3^−/−^→LDLR^−/−^ mice

**Figure 5:**
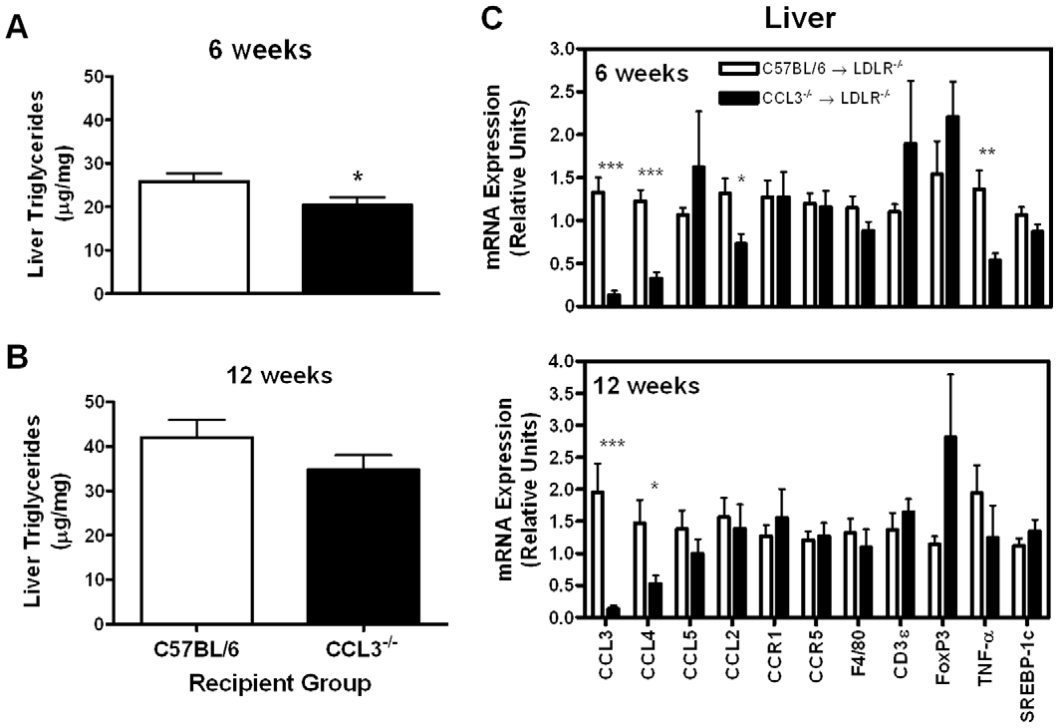
Lipid accumulation and gene expression in liver. Liver was collected from the male mice fed WD for 6 or 12 weeks. (A–B) Liver TG was quantified by GC-MS as described in the Methods section. (C) RNA was isolated and used for real-time RT-PCR analysis as described in the Methods section. Data are the mean ± SEM of the relative gene expression for 8–12 mice per group. CCL2 = monocyte chemoattractant protein-1 TNF-α = tumor necrosis factor-α SREBP-1c = sterol regulatory element binding protein-1c * *P*<0.05 ** *P*<0.01 *** *P*<0.001.

Hepatic TG content after 6 and 12 weeks of WD feeding was reduced by about 20% in male CCL3^−/−^→LDLR^−/−^ mice compared to C57BL/6→LDLR^−/−^ controls (Figure 5A–B). There were no differences in liver TG between the two female groups (data not shown).

Expression levels of chemokines, macrophage markers, and lipogenic genes were also assessed in the livers of BMT mice (Figure 5C). Similar to what was found in adipose tissue, CCL3 expression was nearly undetectable in the CCL3^−/−^→LDLR^−/−^ livers and CCL4 expression was significantly reduced (*P*<0.05). CCL5 expression was not different between recipient groups. TNF-α expression was significantly lower in CCL3^−/−^ recipients at 6 weeks (*P*<0.01). In contrast to the atherosclerotic lesions, T cell markers CD3ε and FoxP3 were not different in livers between groups.

#### Impact of CCL3 on hematopoietic stem cell (HSC) progenitors and leukocytes

**Figure 6:**
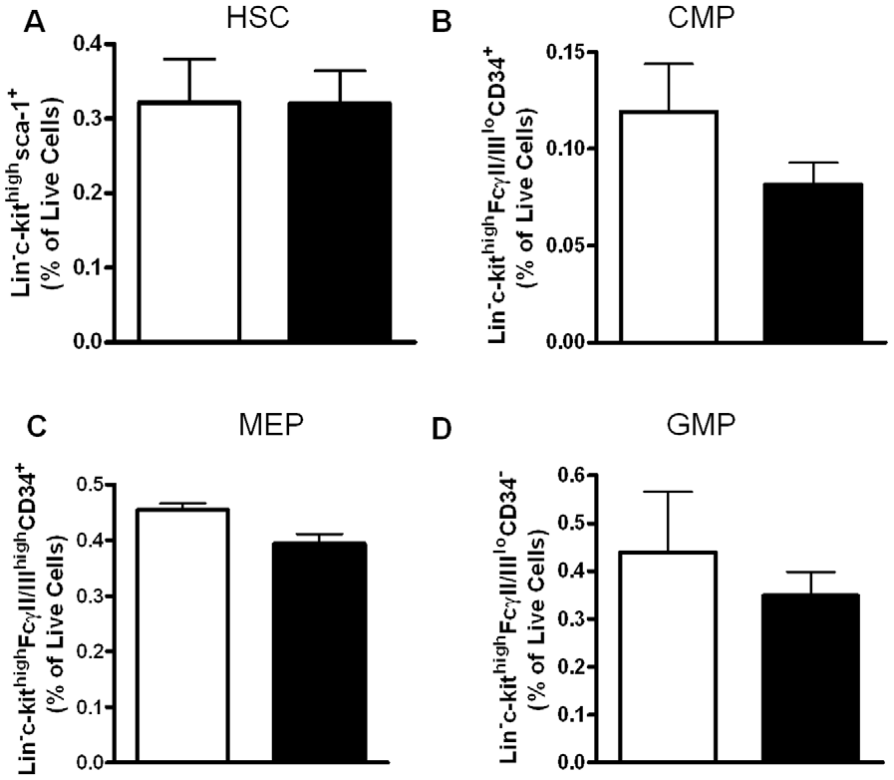
Stem cell progenitors in CCL3^−/−^→LDLR^−/−^ mice. Male LDLR^−/−^ mice were transplanted with C57BL/6 or CCL3^−/−^ bone marrow. At 6 weeks post-WD, bone marrow cells were collected and analyzed by flow cytometry. (A) Quantification of flow cytometry analysis of HSC cells which were identified as lineage negative (Lin^−^) and expressing high levels of c-kit and Sca-1 (Lin^−^c-kit^high^Sca-1^+^). (B) CMP cells were identified as Lin^−^c-kit^high^CD34^+^FcγRII/III^lo^. (C) MEP cells were defined as Lin^−^c-kit^high^CD34^−^FcγRII/III^lo^. (D) GMP cells were defined as Lin^−^c-kit^high^CD34^+^FcγRII/III^high^. Data represent the mean± SEM of 5–6 mice per group. *P<0.05 compared to C57BL/6^−/−^ groups.

Due to the metabolic improvements in the CCL3^−/−^→LDLR^−/−^ mice, we sought to determine whether absence of CCL3 influences reconstitution of hematopoietic stem cells (HSC) and progenitor cells in the bone marrow of LDLR^−/−^ mice after BMT. It is known that injection of CCL3 into mice inhibits progenitor cell proliferation [16]; however, mice globally deficient in CCL3 have no abnormalities in progenitor cells or leukocytes [17]. Bone marrow cells were collected from male recipients of C57BL/6 and CCL3^−/−^ at 6 weeks post-WD and analyzed by flow cytometry for the presence of cell markers expressed on hematopoietic stem cells (HSC) or progenitor cells. HSC were characterized as negative for lineage markers (Lin^−^) and expressed stem cell antigen 1 (Sca-1) and c-kit (for gating strategy, please see Figure S4). There were no differences detected in HSC levels between groups (Figure 6A). Next we examined progenitor cell populations in bone marrow. HSC proliferate and differentiate into progenitor cells, such as common myeloid progenitors (CMP), megakaryocyte/erythroid progenitors (MEP), and granulocyte/macrophage progenitors (GMP), which develop into mature effector cells. Progenitor cells were identified by bone marrow cells expressing c-kit^high^ and CD34 or FcγII/III. There were no significant changes detected in common myeloid progenitor (CMP; Figure 6B) cells, megakaryocyte/erythroid progenitors (MEP; Figure 6C) or granulocyte/macrophage progenitors (GMP; Figure 6D) between groups.

Based on the ability of stem cells to differentiate into various cell types (granulocytes, macrophages, dendritic cells, etc) we examined leukocyte populations in bone marrow and blood at 5 weeks post-BMT and blood, bone marrow, liver, and adipose tissue in mice following 6 weeks of WD. A complete blood count revealed no differences in leukocyte populations (Figure S5, Panel A). With regards to gene expression on blood leukocytes, as expected, CCL3 gene expression was significantly reduced in recipients of CCL3^−/−^ bone marrow (Figure S5, Panel B). However, there were no differences in CCR1, CCR5, or CCL5 expression. Also no differences in lymphocyte or monocyte populations in bone marrow, liver, or adipose tissue were detected following 6 weeks of WD (Figure S6).

### STUDY 2

#### Metabolic parameters in CCL2^−/−^→LDLR^−/−^ mice

To determine whether amelioration of hyperlipidemia and adiposity in BMT LDLR^−/−^ mice was specific to CCL3 deficiency, we performed a second BMT study by transplanting C57BL/6, CCL3^−/−^, or CCL2^−/−^ donor marrow into male LDLR^−/−^ recipients (Study 2 in Figure S1). Four weeks post-BMT, the mice were placed on WD for 6 weeks. The CCL3^−/−^→LDLR^−/−^ mice displayed a similar reduction in body weight, total body adipose tissue, TC, TG, NEFA, and leptin as was seen in the original study (Table 3). However, CCL2^−/−^→LDLR^−/−^ mice were not different from C57BL/6→LDLR^−/−^ mice for any parameter tested. Differences in food intake did not account for the reduced body weight and adiposity in CCL3^−/−^→LDLR^−/−^ mice, resulting in reduced feeding efficiency in these mice (Figure S7).

**Table 3:** Plasma parameters in C67BL/6→LDLR^−/−^, CCL3^−/−^→LDLR^−/−^, and CCL2^−/−^→LDLR^−/−^ mice at 6 weeks post WD-feeding.

Genotype	n	Body weight (g)	Total Body adipose tissue (g)	TC (mg/dL)	TG (mg/dL)	NEFA (mEq/L)	Insulin (ng/mL)	Leptin (ng/mL)
**C57BL/6→LDLR^−/−^**	7	24.1±0.7	3.04±0.38	723±64	263±31	0.82±0.05	0.64±0.13	9.4±2.1
**CCL3^−/−^→LDLR^−/−^**	6	22.2±0.8	2.50±.032	517±55	155±19	0.62±0.09	0.68±0.12	6.1±1.5
**CCL2^−/−^→LDLR^−/−^**	7	24.2±1.0	3.19±0.43	798±103	244±39	0.74±0.07	0.55±0.08	9.6±2.1

Male LDLR^−/−^ mice were transplanted with C57BL/6, CCL3^−/−^ or CCL2^−/−^ bone marrow. At 4 weeks post-BMT, mice were placed on WD for 6 weeks. Data are the mean ± SEM of 6–7 mice per group.

### STUDY 3

#### Body weight, lipoprotein metabolism and atherosclerosis in CCL3^−/−^;LDLR^−/−^ mice

Because we detected reductions in plasma lipids and lesion areas in the BMT model, we investigated whether these reduction were specific to the BMT model. To this end, we developed a double knockout model of CCL3 and LDLR. Male littermate CCL3^+/+^;LDLR^−/−^, CCL3^+/−^;LDLR^−/−^, and CCL3^−/−^; LDLR^−/−^ mice were placed on WD at 8 wks of age and maintained on this diet for 12 weeks (Study #3 in Figure S1). There were no differences among genotypes in body weight throughout the study (Figure S8, Panel A). In addition, adiposity, adipose tissue weight and liver weight were not different between groups (Table S1). Following 12 weeks on WD, there were no differences in plasma parameters among the groups (Table S1). FPLC analyses of plasma cholesterol and triglycerides revealed only modest differences between groups (Figure S8, Panels C and D). Plasma glucose, insulin, and leptin concentrations were unaffected by CCL3 deficiency (Table S2). Similarly, atherosclerotic lesion area was quantified at the aortic root and no differences based on CCL3 genotype were detected (Figure S8, Panel B). Similar results were found for female mice (data not shown).

## Discussion

In this study, we have shown an interesting dichotomy that deficiency of CCL3 in bone marrow-derived cells is protective against hyperlipidemia, atherosclerosis, diet-induced obesity and hepatic steatosis; however, global deficiency of CCL3 in LDLR^−/−^ mice seems to have little impact on these metabolic processes. Furthermore, transplantation of LDLR^−/−^ mice with CCL2^−/−^ marrow does not recapitulate the effects of CCL3. Combined, these data suggest that CCL3 influences bone marrow reconstitution in ways that ultimately impact lipid metabolism in hyperlipidemic mice. In an effort to determine mechanisms by which this occurs, we analyzed bone marrow progenitor stem cell lineages, circulating, and tissue specific immune cell populations. However, CCL3 deficiency did not impact any of these immune cell populations. Taken together these data provide evidence that CCL3 may be an important link in metabolic processes associated with hyperlipidemia; however, the mechanism by which this occurs is not clear.

The primary endpoint of our study was atherosclerotic lesion formation. Previous studies have demonstrated the presence of CCL3 in both mouse and human atherosclerotic lesions [4], [5], [18]; however, a direct role of CCL3 in atherosclerotic lesion formation has not yet been demonstrated. We detected significant reductions in aortic root lesion area in the CCL3^−/−^→LDLR^−/−^ compared to C57BL/6→LDLR^−/−^ mice (Figure 1). The protection from lesion formation in the CCL3^−/−^→LDLR^−/−^ mice is likely due to the reduced plasma lipids, especially because CCL3^−/−^;LDLR^−/−^ mice did not have differences in plasma lipids or atherosclerosis compared to CCL3^+/+^;LDLR^−/−^ mice. The two receptors for CCL3 are CCR1 and CCR5. CCR5^−/−^ mice are protected against atherosclerosis [19], [20], [21] while CCR1^−/−^ mice have accelerated lesion formation [20], [22]. Notably, expression of CCR1 and CCR5 were not different on leukocytes from CCL3^−/−^→LDLR^−/−^ compared to CCL3^+/+^;LDLR^−/−^ mice (Figure S5, Panel B). Thus, absence of a ligand that activates both of these receptors may offset the pro- and anti-atherogenic effects of each.

The observation that deficiency of bone marrow-derived CCL3 leads to a reduction of plasma lipids is a salient finding, although the mechanisms by which this occurs are unclear. A role for chemokines in modulating lipid levels has not been previously reported. For example, changes in CCL2 expression via global knockout or transgenic over-expression do not influence plasma lipid levels [2], [23]. Likewise, deficiency of chemokine receptors CCR1, CCR2, and CCR5 in mice have not been shown to influence lipoprotein metabolism [19], [20], [22], [24], [25]. However, there is ample evidence that the converse is biologically significant, i.e., lipid levels can modulate chemokine expression. We have previously shown that exposure of macrophages to VLDL induces a 5-fold increase in CCL3 gene expression *in vitro* [8]. Human studies also support the notion that CCL3 expression and secretion can be modulated by lipids. In studies of patients with familial hypercholesterolemia, Holven *et al.* have shown that peripheral blood mononuclear cells (PBMCs) from hypercholesterolemic patients had enhanced CCL3, CCL4, and IL-8 expression compared to normolipidemic controls and that release of these chemokines correlated positively with plasma cholesterol levels [26]. This data is supported by the observations of Wahre *et al.* that patients with coronary artery disease had dramatic increases in PBMC chemokine and chemokine receptor expression levels [27], [28], and that the elevated expression was blunted following treatment with statins [27]. Even more interesting, Funk and colleagues made the observation that among 18 inflammatory cytokines and chemokines analyzed, CCL3 was the only one to be consistently increased in plasma of hyperlipidemic mice [10]. The improvements in plasma lipids might be explained by the reduced hepatic steatosis in recipients of CCL3^−/−^ bone marrow. It has previously been shown that CCL2 and CCL3 expression are increased in human steatotic livers [12]. Similarly, plasma CCL3 levels are elevated in high fat fed mice with steatotic livers [29]. Finally, studies by Wouters et al. demonstrated an 11-fold increase in hepatic CCL3 expression in WD-fed LDLR^−/−^ mice whereas C57BL/6 mice had only a 3-fold increase [9]. Thus, it is possible that CCL3 and hyperlipidemia are mechanistically linked.

Hyperlipidemia induces profound effects on immune cell homeostatsis, by enhancing hematopoiesis, monocytosis, and neutrophilia [30], [31], [32]. An alternative to the concept that CCL3 directly influences lipoprotein metabolism is that it indirectly influences lipid homeostasis by modulation of hematopoiesis. It is possible that chemokines within the bone marrow may mediate some of the effects of hyperlipidemia on hematopoiesis In fact, a role for CCL3 in bone marrow myeloid progenitor cell colony formation has been shown *in vitro*, whereby, CCL3 enhances the myelopoietic activity of mature progenitors but suppresses the myelopoietic activity of immature progenitors [33]. Although global CCL3^−/−^ mice were reported to have normal hematopoiesis and leukocyte populations [17]; the ability of these cells to proliferate rapidly to reconstitute a lethally irradiated hyperlipidemic mouse had not been determined. Our studies demonstrate that stem cell lineages were not altered in the bone marrow of CCL3^−/−^ recipients at 6 weeks post-WD. Thus, changes in hematopoiesis following BMT are not likely to be the explanation for the reduced plasma lipid levels and body weight in the CCL3^−/−^→LDLR^−/−^ mice.

Our laboratory is interested in macrophage infiltration not only into the artery wall, but also into adipose tissue. Based on analysis of macrophage markers such as CD68 and F4/80 and inflammatory markers such as TNFα, CCL3^−/−^→LDLR^−/−^ adipose tissue had only slightly lower macrophage content compared to C57BL/6→LDLR^−/−^ recipients. In fact, other data from our laboratory shows that even global deficiency of CCL3 does not reduce adipose tissue macrophage numbers [34]. This was unexpected given CCL3 is highly expressed in adipose tissue [14], [15], [35], and is a potent chemoattractant molecule. Although chemokines have been proposed to be primarily responsible for macrophage recruitment to adipose tissue, and some evidence exists that CCL2 and its receptor, CCR2, contribute to adipose tissue macrophage content [36], [37], conflicting reports have also been published [38], [39], [40]. We also found that CCL4 expression was significantly reduced in CCL3^−/−^→LDLR^−/−^ mice. Thus, the CCL3^−/−^→LDLR^−/−^ mice were effectively deficient in adipose tissue expression of CCL3 and CCL4, and yet adipose tissue macrophage recruitment was not affected. Many studies, including our current report, support a role for individual chemokines in macrophage recruitment to atherosclerotic lesions; however, their role in macrophage recruitment to adipose tissue is more subtle, suggesting more extensive overlap of chemokine function with regards to macrophage recruitment to adipose tissue.

In conclusion, our current data demonstrate reduced body weight, hepatic steatosis, and plasma insulin levels in CCL3^−/−^→LDLR^−/−^ compared to C57BL/6→LDLR^−/−^ mice. This metabolic impact of CCL3 deficiency following BMT is unique to CCL3 as recipients of CCL2^−/−^ marrow did not show the same phenotype. Furthermore, CCL3 deficiency did not impact on lipid metabolism in the double knockout mouse model (CCL3^−/−^;LDLR^−/−^). Although we detected no differences in bone marrow hematopoietic precursors or circulating leukocyte populations following BMT, our combined data point to a role for CCL3 in metabolism following bone marrow reconstitution of hyperlipidemic LDLR^−/−^ mice.

## Materials and Methods

### Mice

All animal care procedures were performed in accordance with and approval by the Vanderbilt University Institutional Animal Care and Use Committee (approval # M06/476). C57BL/6, LDLR^−/−^, CCL3^−/−^, and CCL2^−/−^ mice were originally purchased from Jackson Laboratories (Bar Harbor, ME) and were propagated within our colony to produce the donor and recipient mice used in this study. All mice were on the C57BL/6 background. **Study 1:** At 8 weeks of age, mice were lethally irradiated with 900 rads from a Cesium gamma source and transplanted with marrow from C57BL/6 or CCL3^−/−^ marrow collected from the long bones of donor mice. Hereafter, these groups will be referred to as C57BL/6→LDLR^−/−^ and CCL3^−/−^→LDLR^−/−^. Recipient mice were given water containing 0.25% neomycin/polymyxin B sulfate (Monarch Pharmaceuticals, Bristol, TN) for 1 week prior to and 2 weeks following bone marrow transplantation (BMT). Four weeks post-BMT, mice were *ad libitum* fed Western diet (WD) which contains 42% of kcal from milk fat with 0.15% cholesterol added (Harlan-Teklad, Madison, WI) for 6 or 12 weeks (for study design, please see Figure S1). **Study 2:** Male LDLR^−/−^ mice were transplanted with bone marrow from C57BL/6, CCL3^−/−^ or CCL2^−/−^ donors and placed on the WD for 6 weeks. These groups are designated as C57BL/6→LDLR^−/−^, CCL3^−/−^→LDLR^−/−^ and CCL2^−/−^→LDLR^−/−^. **Study 3:** CCL3^−/−^ and LDLR^−/−^ mice were crossed to generate CCL3^+/−^;LDLR^+/−^ mice, which were then bred to generate littermate CCL3^+/+^;LDLR^−/−^, CCL3^+/−^;LDLR^−/−^, and CCL3^−/−^;LDLR^−/−^ mice. At the end of each study, mice were fasted for 5 h, bled via the retro-orbital plexus, perfused with PBS, and tissues collected. Blood was centrifuged, plasma collected, and frozen. Blood was also used for DNA analysis to confirm the complete reconstitution of the BMT mice with donor bone marrow (data not shown).

### Food intake

Mice were housed 2–3 mice per cage, and the weight of the food remaining in the cage was quantified 2 times per week. The average daily food intake per mouse was calculated based on the number of mice per cage and the difference between the starting and ending food weights. Feeding efficiency was calculated by taking the average weight gain in mg/average daily caloric intake.

### Plasma analyses

Total plasma cholesterol (TC) and triglyceride (TG) levels were measured using enzymatic assays from Cliniqa Inc. (San Diego, CA). Very low-density lipoprotein (VLDL), low-density lipoprotein (LDL), and high-density lipoprotein (HDL) were measured by using fast performance liquid chromatography (FPLC) as described [41]. Non-esterified fatty acids (NEFAs) were measured using the NEFA-HR 2 kit from Wako (Richmond, VA). Insulin and leptin levels were measured by RIA in the Hormone Assay Core Laboratory of the Vanderbilt Mouse Metabolic Phenotyping Center (MMPC).

### Body composition analysis

Mice were analyzed for total body adipose tissue and lean body mass before transplantation, 4 weeks post-BMT (before starting WD feeding), 6 weeks-post WD, and 12 weeks post-WD. These analyses were performed by nuclear magnetic resonance using the Bruker Minispec (Woodlands, TX) at the Vanderbilt University MMPC.

### Atherosclerotic lesion formation

At sacrifice, hearts from all mice were placed in OCT and frozen on dry ice. Hearts were cut according to the method of Paigen *et al.* [42] and 15 sections extending from the aortic root for 300 µm were collected. Sections were stained with Oil Red O (ORO), images captured using a Q-imaging Micropublisher camera mounted on an Olympus upright microscope, and the stained area quantified using Histometrix 6 software by Kinetic Imaging, Ltd. (Durham, NC).

### Immunohistochemical Analysis

Five µm aortic root sections were stained for MOMA using 1∶50 dilution of rat anti-mouse macrophage antibody from Serotec (Raleigh, NC). Briefly, sections were in cold acetone and washed with PBS. Sections were then blocked in 2% BSA/PBS for 30 min. A 15 min avidin/biotin block (SP-2001 Vector Laboratories Burlingame, CA) was performed. For CD4 immunofluorescence, rat anti-mouse CD4 primary antibody (BD Biosciences, San Jose, CA) was applied at 1∶20 dilution for 1 h, slides were washed with PBS and secondary antibody (biotinylated goat anti-rat IgG) was applied at 1∶100 for 30 min at 37°C. Strepavidin- Texas Red (A-2006 Vector Laboratories Burlingame, CA) was applied at 10 mg/ml in 2% BSA/PBS for 30 min and washed with PBS. Vectorshield (H-1200 Vector Laboratories, Burlingame, CA) was added and slides were coverslipped.

### RNA isolation and realtime RT-PCR

RNA was isolated from 100 mg of perigonadal adipose tissue using the RNeasy minikit from Qiagen (Valencia, CA). cDNA was synthesized using the iScript cDNA synthesis kit from BioRad (Hercules, CA). cDNA was diluted 1∶5 or 1∶10 and then used for real-time PCR analysis on a BioRad iQ5 machine. Primer-probe sets were purchased from the “Assays-on-Demand” program from Applied Biosystems (Foster City, CA). Quantification of 18S was performed for each sample, and final relative concentration was determined by comparing each gene of interest to 18S using the delta delta CT method [43].

### Adipose tissue histochemistry

Perigonadal adipose tissue was fixed in 10% formalin overnight and then embedded in paraffin. Paraffin sections were fixed and stained with Toluidine Blue O (TBO) according to manufacturer's instructions (Newcomer Supply, Middleton, WI).

### Flow Cytometry

Cells isolated from various tissue were first incubated with Fc block for 5 min at room temperature, followed by incubation for 20 min at 4°C with fluorophore conjugated antibodies: F4/80-APC (eBioscience), Ly6C-FITC (BD Biosciences), CD11b-APC-Cy7 (BD Bioscience), Ly6G-PE (BD Bioscience), Lineage marker-Alexa488 (Molecular Probes), FcγRII/III-PE-Cy7 (BD Bioscience), CD34-Alexa647 (BD Bioscience), Sca-1-Pacific Blue (BioLegend), and c-kit-PE (eBiosciences). Samples were processed on a 5 Laser LSRII machine in the Vanderbilt Flow Cytometry Core and data analyzed using FlowJo software.

### Statistical analysis

Comparisons between recipients of C57BL/6 and CCL3^−/−^ mice at each time point were performed using un-paired Student's *t*-tests. Comparisons among C67BL/6→LDLR^−/−^, CCL3^−/−^→LDLR^−/−^, and CCL2^−/−^→LDLR^−/−^ transplant groups were performed using one-way ANOVA with a Tukey post-hoc test. Comparisons among CCL3^+/+^;LDLR^−/−^, CCL3^+/−^;LDLR^−/−^,and CCL3^−/−^;LDLR^−/−^ groups were also performed using one-way ANOVA with a Tukey post-hoc test.

## Supporting Information

Figure S1 **Experimental Design.** **Study #1:** Eight week old male and female LDLR^−/−^ mice were lethally irradiated and reconstituted with bone marrow collected from C57BL/6 or CCL3^−/−^ donors. Two separate cohorts were transplanted 6 weeks apart and placed on WD for either 6 or 12 weeks starting 4 weeks after their respective transplantations. **Study #2:** Eight week old male LDLR^−/−^ mice were lethally irradiated and reconstituted with bone marrow collected from C57BL/6, CCL3^−/−^ or MCP-1^−/−^ donors. Four weeks after transplantation, mice were fed WD for 6 weeks. **Study #3:** Eight week old littermate CCL3^+/+^;LDLR^−/−^, CCL3^+/−^;LDLR^−/−^, and CCL3^−/−^;LDLR^−/−^ were placed on WD for 12 weeks.
(TIF)

Figure S2 **Glucose tolerance test and plasma insulin levels.** LDLR^−/−^ mice were transplanted with C57BL/6 or CCL3^−/−^ bone marrow. At four weeks post-BMT, mice were placed on WD for 6 weeks. (A) Mice were fasted for 5 h and basal blood glucose levels were measured (0 min) before intraperitoneal administration of 1.5 g glucose per kg lean body mass. Blood glucose was assessed at 15, 30, 45, 60, 90, and 150 min after injection. (B) Plasma insulin levels were measured 30 min after administration of the glucose bolus. Data are the mean ± SEM of 3–6 mice per group.
(TIF)

Figure S3 **FPLC analysis of lipoprotein profiles.** LDLR^−/−^ mice were transplanted with C57BL/6 or CCL3^−/−^ bone marrow. At four weeks post-BMT, mice were placed on WD for 6 or 12 weeks. Plasma lipids were fractionated and cholesterol quantified as described in the Methods section. A) females at 6 weeks post-WD; B) males at 6 weeks post-WD; C) females at 12 weeks post-WD; D) males at 12 weeks post-WD.
(TIF)

Figure S4 **Gating strategy for bone marrow HSC and progenitor cells.** LDLR^−/−^ mice were transplanted with C57BL/6 or CCL3^−/−^ bone marrow. At 5 weeks post-BMT, bone marrrow cells were collected from recipient mice and analyzed by flow cytometry. A) HSC cells were identified as lineage negative cells (Lin^−^) cells and expression of Sca-1 and c-kit (Lin^−^c^−^kit^high^Sca-1^+^). B) Plot of progenitor cells from C57BL/6 bone marrow cells were identified by expressing c-kit^high^Sca-1^−^ and CD34 or FcγRII/III. CMP cells were defined as Lin^−^c-kit^high^CD34^+^FcγRII/III^lo^. MEP cells were defined as Lin^−^c-kit^high^CD34^−^FcγRII/III^lo^. GMP cells were defined as Lin^−^c-kit^high^CD34^+^FcγRII/III^high^. C) Plot of progenitor cells in CCL3^−/−^ bone marrow cells. Data presented are representative plots of the gating strategy for HSC and progenitor cells.
(TIF)

Figure S5 **Complete blood counts and gene expression.** LDLR^−/−^ mice were transplanted with C57BL/6 or CCL3^−/−^ bone marrow. At four weeks post-BMT, mice were placed on WD for 6 weeks. A) Blood was collected and a complete blood cell was performed. NE = Neutrophils, LY = Lymphocytes, MO = monocytes, EO = Eosinophils, BA = Basophils. B) Leukocytes were collected from blood and RNA was isolated and analyzed by real-time PCR as described in the Methods section. Data are the mean ± SEM of the relative gene expression for 3–6 mice per group.
(TIF)

Figure S6 **Leukocyte populations in adipose tissue, bone marrow, and liver.** LDLR^−/−^ mice were transplanted with C57BL/6 or CCL3^−/−^ bone marrow. At 6 weeks post-BMT cells were collected from adipose tissue, bone marrow, and liver. Cells were then analyzed by flow cytometry. A–B) Adipose tissue T cells and macrophages; C–D) Bone marrow monocytes and neutrophils; E–F) Liver T cells and monocytes. T cells were identified by expressing TCRb. Macrophages were identified by expressing CD11b^+^F4/80^+^. Monocytes were identified by expressing CD11b^+^Ly6C^+^Ly6G^−^. Neutrophils were identified by expressing CD11b^+^Ly6C^+^Ly6G^+^. Data are the mean ± SEM of 3–6 mice per group.
(TIF)

Figure S7 **Food intake and feeding efficiency.** Daily food intake and feeding efficiency were quantified for the mice in Study #2 according to the Methods section. Data are the mean ± SEM of 6–7 mice per group.
(TIF)

Figure S8 **Body weight, atherosclerosis, and plasma lipoprotein profiles in CCL3^−/−^;LDLR^−/−^ mice.** Male CCL3^−/−^;LDLR^−/−^ were started on WD at 8 weeks of age and maintained on the diet for 12 weeks. A) Body mass during WD feeding. B) Aortic root lesion quantification at 12 weeks post-WD. Data are from 5–8 mice per group. C and D) FPLC analysis of lipoprotein profiles of mice at 12 weeks post WD-feeding. Plasma lipids were fractionated and cholesterol (C) and triglycerides (D) were quantified as described in the Methods section.
(TIF)

Table S1 **Body and tissue masses after 12 weeks of Western diet feeding.** CCL3^+/+^;LDLR^−/−^, CCL3^+/−^;LDLR^−/−^, and CCL3^−/−^;LDLR^−/−^ mice were placed on WD for 12 weeks. Body weight as well as total body lean and fat mass and liver mass were measured. Data are the mean ± SEM from the number of mice indicated.
(DOC)

Table S2 **Plasma parameters in CCL3^+/+^;LDLR^−/−^, in CCL3^+/−^;LDLR^−/−^, and CCL3^−/−^;LDLR^−/−^, after 12 weeks of Western diet feeding.** CCL3^+/+^;LDLR^−/−^, CCL3^+/−^;LDLR^−/−^, and CCL3^−/−^;LDLR^−/−^ mice were placed on WD for 12 weeks. Plasma parameters were measured from blood collected as described in the Methods section. Data are the mean ± SEM from the number of mice indicated.
(DOCX)
