# Supplementary figures and images for: Impact of Macrophage Inflammatory Protein-1α Deficiency on Atherosclerotic Lesion Formation, Hepatic Steatosis, and Adipose Tissue Expansion
Source: PLoS One. 2012 Feb 16;7(2):e31508. doi: 10.1371/journal.pone.0031508 (PMC3281060; doi:10.1371/journal.pone.0031508)

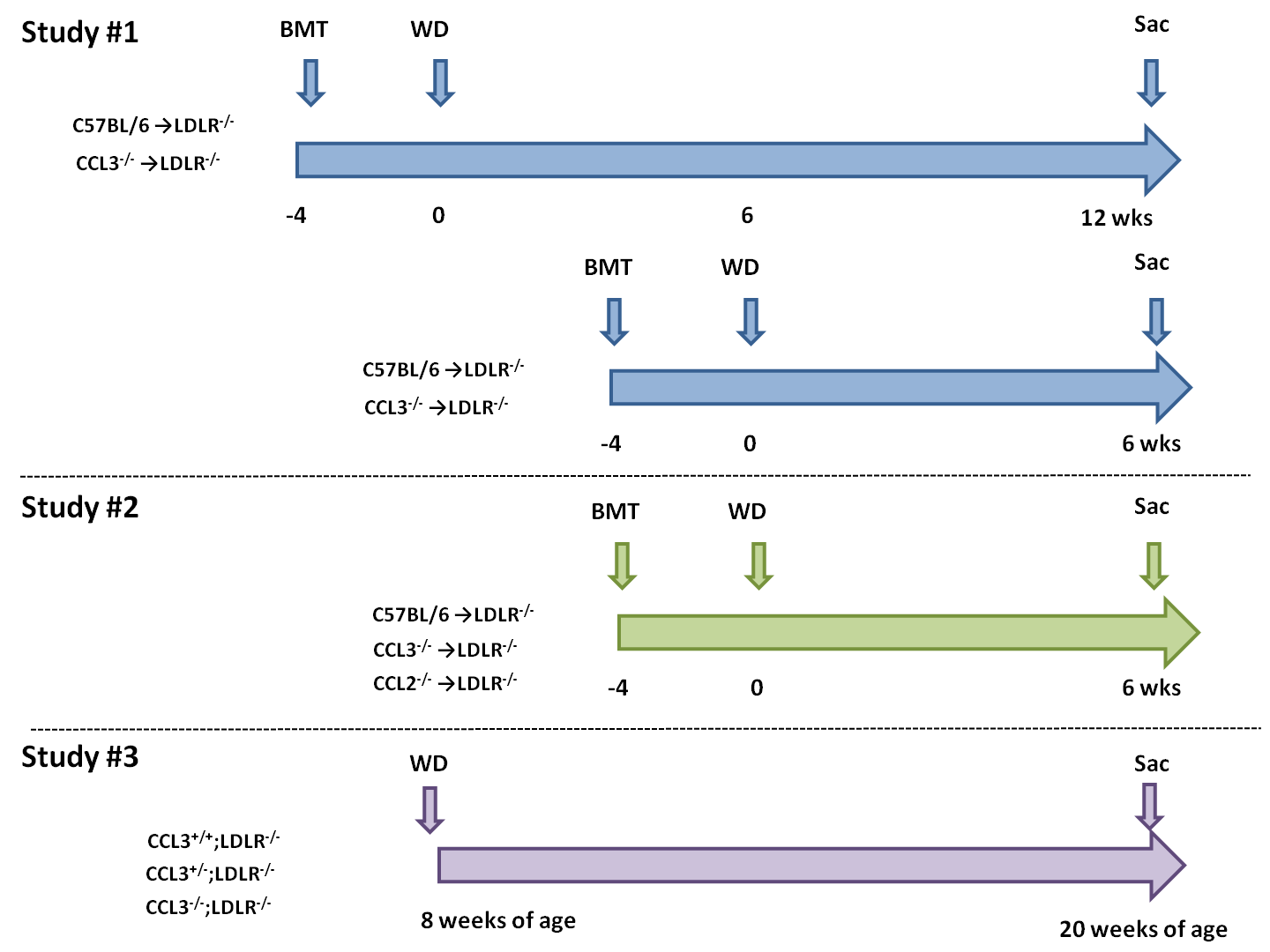

Supplement: Figure S1 — Experimental Design. Study #1: Eight week old male and female LDLR−/− mice were lethally irradiated and reconstituted with bone marrow collected from C57BL/6 or CCL3−/− donors. Two separate cohorts were transplanted 6 weeks apart and placed on WD for either 6 or 12 weeks starting 4 weeks after their respective transplantations. Study #2: Eight week old male LDLR−/− mice were lethally irradiated and reconstituted with bone marrow collected from C57BL/6, CCL3−/− or MCP-1−/− donors. Four weeks after transplantation, mice were fed WD for 6 weeks. Study #3: Eight week old littermate CCL3+/+;LDLR−/−, CCL3+/−;LDLR−/−, and CCL3−/−;LDLR−/− were placed on WD for 12 weeks. (TIF) [file pone.0031508.s001.tif]

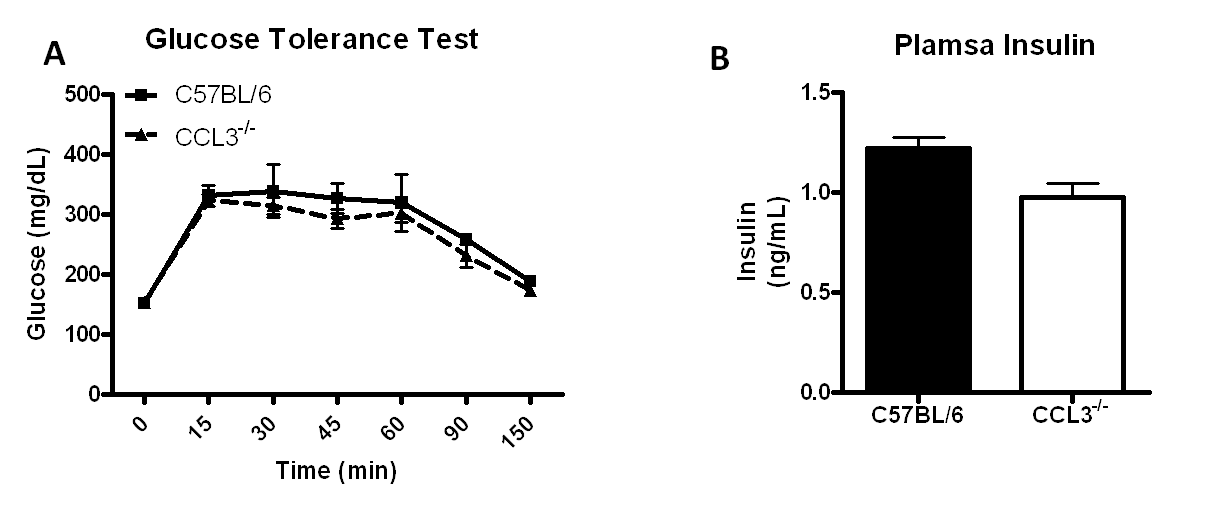

Supplement: Figure S2 — Glucose tolerance test and plasma insulin levels. LDLR−/− mice were transplanted with C57BL/6 or CCL3−/− bone marrow. At four weeks post-BMT, mice were placed on WD for 6 weeks. (A) Mice were fasted for 5 h and basal blood glucose levels were measured (0 min) before intraperitoneal administration of 1.5 g glucose per kg lean body mass. Blood glucose was assessed at 15, 30, 45, 60, 90, and 150 min after injection. (B) Plasma insulin levels were measured 30 min after administration of the glucose bolus. Data are the mean ± SEM of 3–6 mice per group. (TIF) [file pone.0031508.s002.tif]

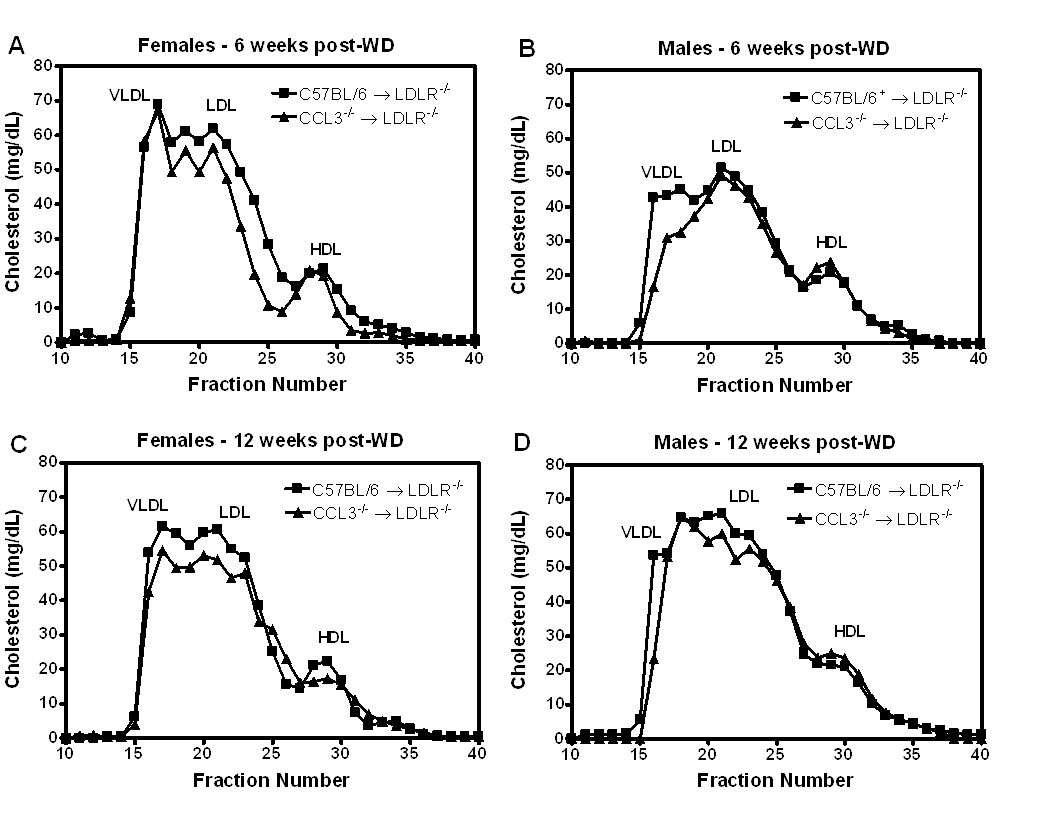

Supplement: Figure S3 — FPLC analysis of lipoprotein profiles. LDLR−/− mice were transplanted with C57BL/6 or CCL3−/− bone marrow. At four weeks post-BMT, mice were placed on WD for 6 or 12 weeks. Plasma lipids were fractionated and cholesterol quantified as described in the Methods section. A) females at 6 weeks post-WD; B) males at 6 weeks post-WD; C) females at 12 weeks post-WD; D) males at 12 weeks post-WD. (TIF) [file pone.0031508.s003.tif]

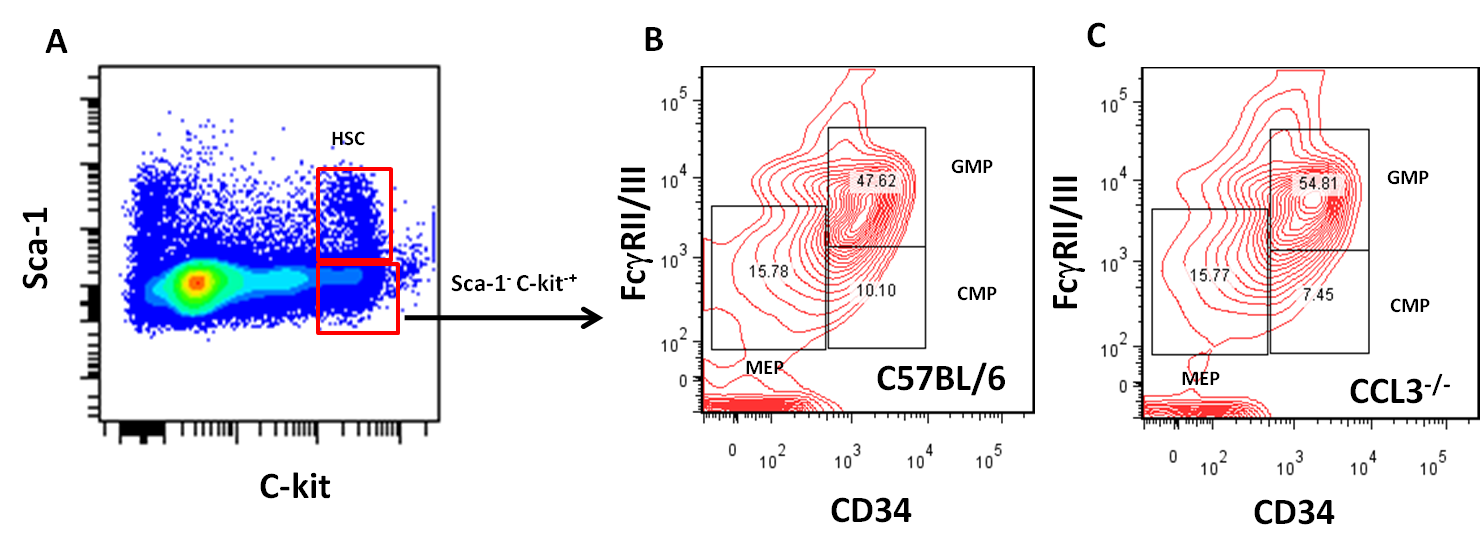

Supplement: Figure S4 — Gating strategy for bone marrow HSC and progenitor cells. LDLR−/− mice were transplanted with C57BL/6 or CCL3−/− bone marrow. At 5 weeks post-BMT, bone marrrow cells were collected from recipient mice and analyzed by flow cytometry. A) HSC cells were identified as lineage negative cells (Lin−) cells and expression of Sca-1 and c-kit (Lin−c−kithighSca-1+). B) Plot of progenitor cells from C57BL/6 bone marrow cells were identified by expressing c-kithighSca-1− and CD34 or FcγRII/III. CMP cells were defined as Lin−c-kithighCD34+FcγRII/IIIlo. MEP cells were defined as Lin−c-kithighCD34−FcγRII/IIIlo. GMP cells were defined as Lin−c-kithighCD34+FcγRII/IIIhigh. C) Plot of progenitor cells in CCL3−/− bone marrow cells. Data presented are representative plots of the gating strategy for HSC and progenitor cells. (TIF) [file pone.0031508.s004.tif]

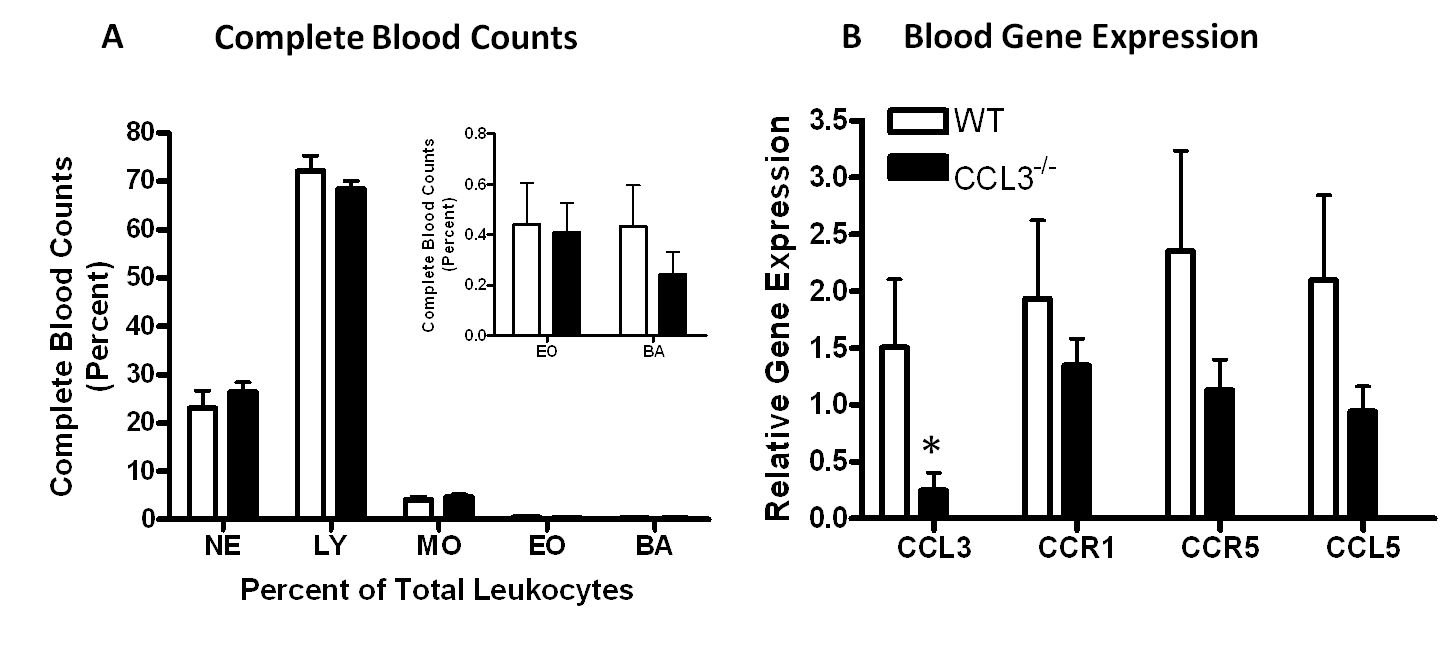

Supplement: Figure S5 — Complete blood counts and gene expression. LDLR−/− mice were transplanted with C57BL/6 or CCL3−/− bone marrow. At four weeks post-BMT, mice were placed on WD for 6 weeks. A) Blood was collected and a complete blood cell was performed. NE = Neutrophils, LY = Lymphocytes, MO = monocytes, EO = Eosinophils, BA = Basophils. B) Leukocytes were collected from blood and RNA was isolated and analyzed by real-time PCR as described in the Methods section. Data are the mean ± SEM of the relative gene expression for 3–6 mice per group. (TIF) [file pone.0031508.s005.tif]

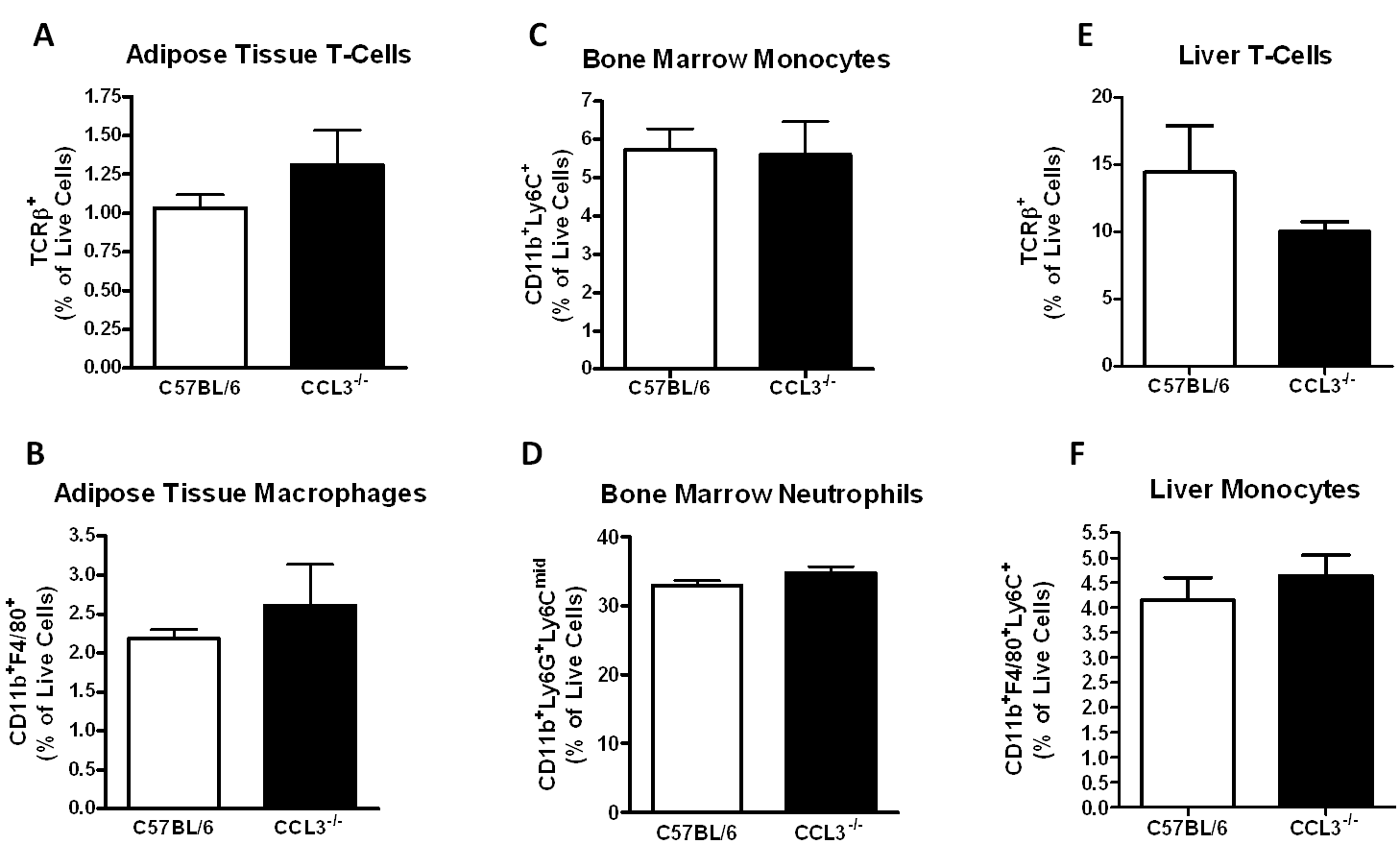

Supplement: Figure S6 — Leukocyte populations in adipose tissue, bone marrow, and liver. LDLR−/− mice were transplanted with C57BL/6 or CCL3−/− bone marrow. At 6 weeks post-BMT cells were collected from adipose tissue, bone marrow, and liver. Cells were then analyzed by flow cytometry. A–B) Adipose tissue T cells and macrophages; C–D) Bone marrow monocytes and neutrophils; E–F) Liver T cells and monocytes. T cells were identified by expressing TCRb. Macrophages were identified by expressing CD11b+F4/80+. Monocytes were identified by expressing CD11b+Ly6C+Ly6G−. Neutrophils were identified by expressing CD11b+Ly6C+Ly6G+. Data are the mean ± SEM of 3–6 mice per group. (TIF) [file pone.0031508.s006.tif]

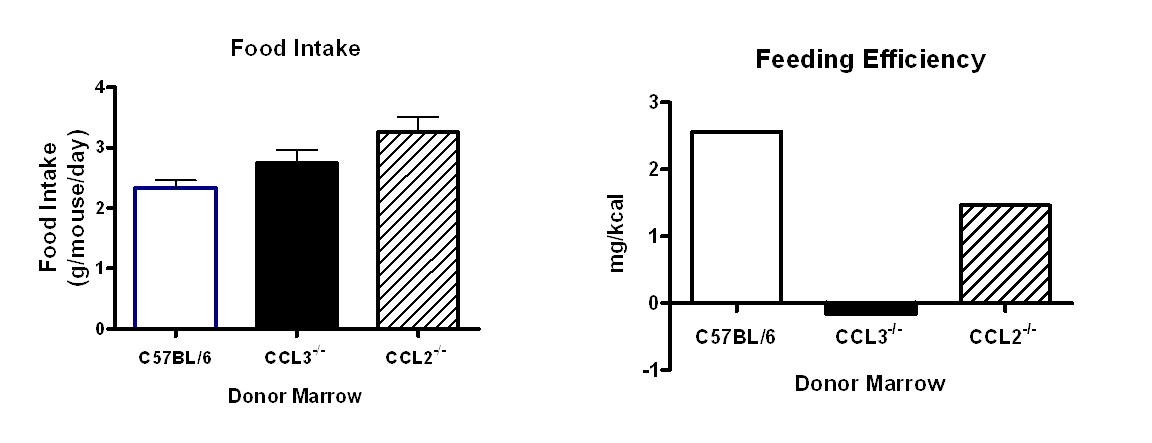

Supplement: Figure S7 — Food intake and feeding efficiency. Daily food intake and feeding efficiency were quantified for the mice in Study #2 according to the Methods section. Data are the mean ± SEM of 6–7 mice per group. (TIF) [file pone.0031508.s007.tif]

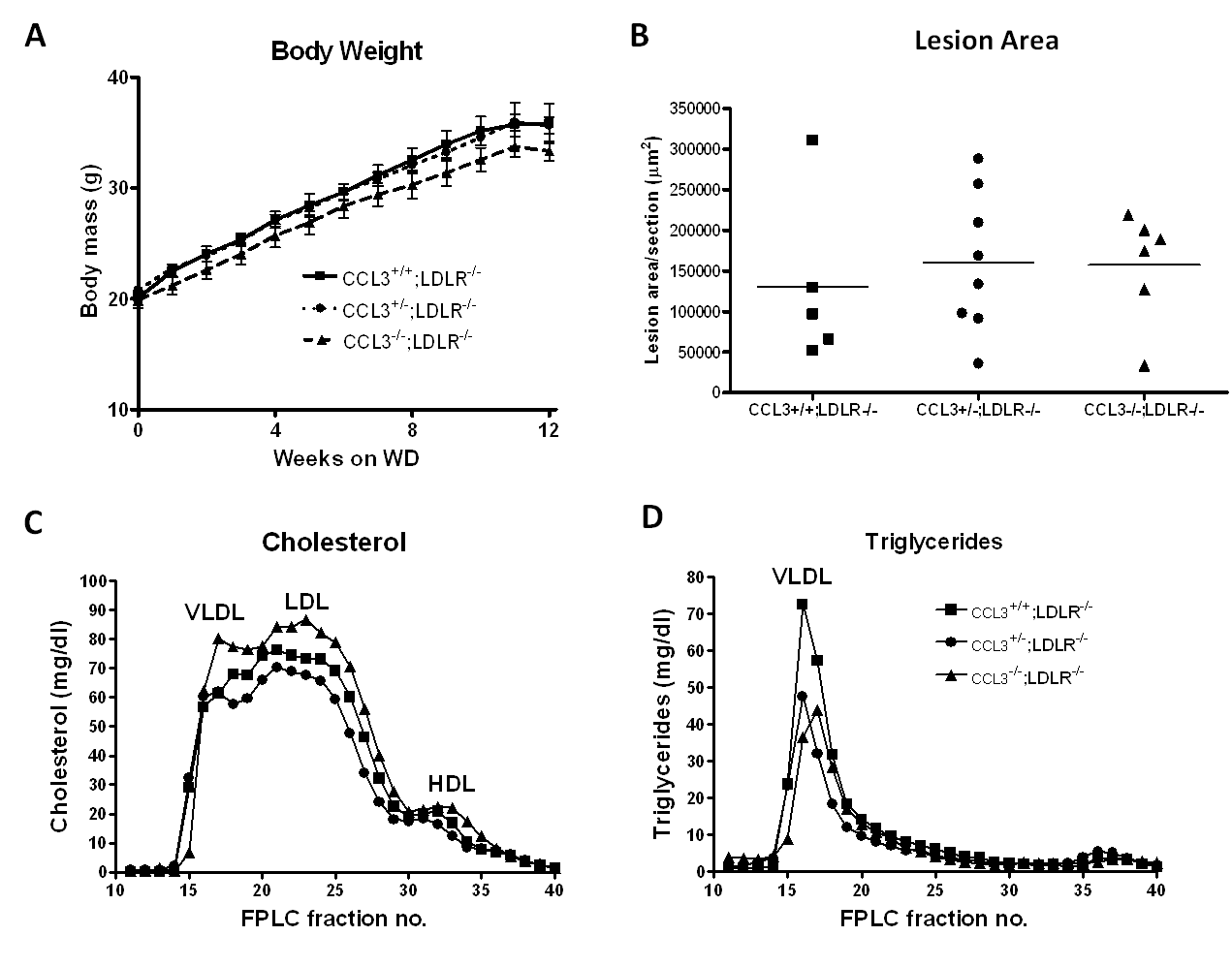

Supplement: Figure S8 — Body weight, atherosclerosis, and plasma lipoprotein profiles in CCL3−/−;LDLR−/− mice. Male CCL3−/−;LDLR−/− were started on WD at 8 weeks of age and maintained on the diet for 12 weeks. A) Body mass during WD feeding. B) Aortic root lesion quantification at 12 weeks post-WD. Data are from 5–8 mice per group. C and D) FPLC analysis of lipoprotein profiles of mice at 12 weeks post WD-feeding. Plasma lipids were fractionated and cholesterol (C) and triglycerides (D) were quantified as described in the Methods section. (TIF) [file pone.0031508.s008.tif]
