# Supplementary material for: Impact of Macrophage Inflammatory Protein-1α Deficiency on Atherosclerotic Lesion Formation, Hepatic Steatosis, and Adipose Tissue Expansion
Source: PLoS One. 2012 Feb 16;7(2):e31508. doi: 10.1371/journal.pone.0031508 (PMC3281060; doi:10.1371/journal.pone.0031508)
Supplement: Table S1 — Body and tissue masses after 12 weeks of Western diet feeding. CCL3+/+;LDLR−/−, CCL3+/−;LDLR−/−, and CCL3−/−;LDLR−/− mice were placed on WD for 12 weeks. Body weight as well as total body lean and fat mass and liver mass were measured. Data are the mean ± SEM from the number of mice indicated. (DOC) [file pone.0031508.s009.doc]

**Table S1.** **Body and Tissue Masses after 12 Weeks of Western Diet Feeding.**

| **Genotype** | **n** | **Body Weight (g)** | **Total WAT**  **Mass (g)** | **Lean Tissue**  **Mass (g)** | **Perigonadal WAT Mass (g)** | **Liver Mass**  **(g)** |
| --- | --- | --- | --- | --- | --- | --- |
| CCL3^+/+^;LDLR^-/-^ | 8 | 34.0 ± 1.5 | 10.3 ± 1.1 | 20.5 ± 0.5 | 1.52 ± 0.15 | 1.74 ± 0.12 |
| CCL3^+/-^;LDLR^-/-^ | 17 | 35.6 ± 0.8 | 10.9 ± 0.5 | 21.6 ± 0.3 | 1.59 ± 0.08 | 1.88 ± 0.08 |
| CCL3^-/-^;LDLR^-/-^ | 12-13 | 33.5 ± 0.9 | 10.2 ± 0.7 | 20.3 ± 0.3 | 1.38 ± 0.10 | 1.87 ± 0.09 |
