# Supplementary material for: Impact of Macrophage Inflammatory Protein-1α Deficiency on Atherosclerotic Lesion Formation, Hepatic Steatosis, and Adipose Tissue Expansion
Source: PLoS One. 2012 Feb 16;7(2):e31508. doi: 10.1371/journal.pone.0031508 (PMC3281060; doi:10.1371/journal.pone.0031508)
Supplement: Table S2 — Plasma parameters in CCL3+/+;LDLR−/−, in CCL3+/−;LDLR−/−, and CCL3−/−;LDLR−/−, after 12 weeks of Western diet feeding. CCL3+/+;LDLR−/−, CCL3+/−;LDLR−/−, and CCL3−/−;LDLR−/− mice were placed on WD for 12 weeks. Plasma parameters were measured from blood collected as described in the Methods section. Data are the mean ± SEM from the number of mice indicated. (DOCX) [file pone.0031508.s010.docx]

**Table S2.** **Plasma Parameters in CCL3^+/+^;LDLR^-/-^, in CCL3^+/-^;LDLR^-/-^, and CCL3^-/-^;LDLR^-/-^, after 12 Weeks of Western Diet Feeding.**

| **Genotype** | **n** | **TC**  **(mg/dl)** | **TG**  **(mg/dl)** | **NEFA**  **(mEq/l)** | **Glucose (mg/dl)** | **Insulin**  **(ng/ml)** | **Leptin**  **(ng/ml)** |
| --- | --- | --- | --- | --- | --- | --- | --- |
| CCL3^+/+^;LDLR^-/-^ | 5-8 | 1291 ± 46 | 396 ± 40 | 3.54 ± 0.26 | 125 ± 4 | 1.35 ± 0.18 | 50.3 ± 10.6 |
| CCL3^+/-^;LDLR^-/-^ | 7-17 | 1139 ± 52 | 332 ± 26 | 3.13 ± 0.12 | 115 ± 4 | 2.27 ± 0.37 | 45.6 ± 6.1 |
| CCL3^-/-^;LDLR^-/-^ | 7-13 | 1255 ± 85 | 322 ± 31 | 3.30 ± 0.35 | 122 ± 4 | 1.29 ± 0.26 | 46.1 ± 9.4 |
